# The Recreational Trail of the El Caminito del Rey Natural Tourist Attraction, Spain: Determination of Hikers’ Flow

**DOI:** 10.3390/ijerph18041809

**Published:** 2021-02-12

**Authors:** Gemma María Gea-García, Carmelo Fernández-Vicente, Francisco J. Barón-López, Jesús Miranda-Páez

**Affiliations:** 1 Department of Sport Science, Faculty of Sport Sciences, San Antonio Catholic University, 30107 Murcia, Spain; 2 Department of Psychobiology and Methodology of Behavioral Sciences, Faculty of Psychology, University of Málaga, 29071 Málaga, Spain; jmpaez@uma.es; 3 Department of Public Health and Psychiatry, Faculty of Health Sciences, University of Málaga, 29071 Málaga, Spain; fjbaron@gmail.com

**Keywords:** outdoor recreation, recreational trails, spatiotemporal analysis, eye tracking, hikers’ flow, hiking management

## Abstract

Hiking is a very popular outdoor activity, and has led to an exponential increase in the number of visitors to natural spaces. The objective of this study was to analyze the circulation pattern of visitors to the Caminito del Rey trail, based on the three zones into which the trail can be divided. The sample consisted of 1582 hikers distributed into three different profiles. Of these, 126 utilized an eye-tracking device during the hike, while, for the rest (1456), only their travel speed along the trail was recorded. The use of eye tracking devices identified a greater number of interesting landscapes located in zones 1 and 3 of the trail, and it was observed that the mean travel speed was greater for zone 2 (42.31 m/min) (*p* < 0.01). Additionally, when the three different visitor profiles were analyzed, significant differences were found between the mean travel speeds according to sectors (*p* < 0.05). This information is crucial for more efficient management of the trail, as it allows for the development of measures to control and regulate the flow of visitors according to zone, and the design of additional strategies to increase the awareness of the hiker about specific areas of the hike.

## 1. Introduction

Recently, nature tourism has become one of the most desired leisure experiences [1,2], which has resulted in the exponential increase in activities performed outdoors [3,4]. Within the great choice of activities that can be performed in a natural environment, hiking should be highlighted, as it is considered one of the most popular leisure activities in the world [2,5,6,7,8].

Collins-Kreiner and Kliot [9] define hiking as a simple way of moving at a slow pace, characterized by the existence of intermittent relationships between people, as well as between people and their environment. According to Mohd-Taher et al. [10], hiking has become a very lucrative business, because tour operators sell complete packages of guided visits in natural areas. This allows greater visibility and access to areas, natural parks, and/or mountain regions, which would otherwise not be available to the public. The access to remote natural areas that have great biodiversity or some type of feature in the landscape to be observed translates into an increase in the number of visits [9,11,12,13,14,15]. This exponential growth of visitors to natural areas can provoke certain problems related to their efficient management and sustainable development [1,16], and can increase the importance of monitoring the hiker’s safety [5,8,15,17,18,19,20].

The increasing popularity of hiking has led scientific research to focus its attention on the study of the existing differences in the spatiotemporal flow of the hikers [1,7,13,14,15,16,21,22,23]. Thus, it is observed that hikers themselves, as well as the surroundings where the sport is practiced, have become priorities in scientific research [1,4,18,24,25,26].

Many research studies have focused on the hiker, and have pointed out that the hiker profile has become unequal, in that situations were observed where many profiles of practitioners of physical sports activities (such as hikers, horseback riders, cyclists, or joggers) were found in natural areas [1,7,8,14,15,16,27]. Furthermore, there are different types or groups of hikers with different objectives or interests [26,28,29], who opt for performing their activity in nature either guided or non-guided, as observed with other sports performed in nature [6,27,30,31]. In light of this, many research studies have stated that it is possible to differentiate between visitor groups with different needs and behaviors throughout the trails [7,8,15,17,23,29]. Therefore, it is necessary to gather the information about the spatiotemporal data of visitor flows [5,19,23], such as the composition of the group of practitioners, type of visit chosen, or time spent [29]. The analysis of these data could help with the efficient and safe management of the area and the hiker [1,13,29]. Moreover, spatial information on hiker flow could be used to direct visitors to different park locations to avoid overcrowding, or away from possible high-risk situations in which the intervention of emergency or rescue personnel is needed [5,8,15,17,18,19,20,32].

To adequately investigate the pattern of movement in any natural landscape, it is necessary to have in mind the physical characteristics of the area itself, as well as the type of trail on which hiking takes place [15,17,21,29,33,34], given that each of these elements will allow for the shaping or identification of what the literature defines as the “relevant place” compared to another that is not [21,35]. Beeco et al. [17] defined trails as the main conduit through which the visitor flows, so that they are considered to be a resource of great value for the visitor experience. The reason is that trails provide an opportunity for accessing nature tourism attractions and limit their use or passage through specific places [8,13,19,23,24], either to reduce the risk of becoming lost, physical danger to the hiker, or damage to sensitive areas [25]. Orellana et al. [21] and Lera et al. [3] define the concept of the “relevant place” as a place where movement suspension patterns are detected, which could be associated with a relevant geographical characteristic of the terrain. Along this line, Chhetrei et al. [15] and Ried et al. [36] affirm that the perception of the landscape is a complex cognitive construction, in which the hikers will perceive the medium that surrounds them and its characteristics as something pleasant or unpleasant. This process of perception will provoke an affective state that is more or less positive, and which will be different for each hiker depending on the expectations or preferences [35,36]. Furthermore, each of the geographical characteristics of the natural landscape will have a different potential value and attractiveness, where certain aspects, such as the density or size of the trees or the presence of water, will have special relevance when catching the attention of the hiker [11,14,15,17,21,29,34,35,36,37]. All of this is in agreement with the ideas postulated in the Attention Restoration Theory (ART). One of the main characteristics of the ART is the phenomenon of fascination, defined as the involuntary attention maintained with little or no effort [35,36,37]. At present, it has been observed how environments with natural elements tend to evoke a greater pleasure, and therefore a greater interest in people, as compared to environments without natural elements, which is without a doubt a trigger of this fascination phenomenon [14,35,36,37]. In light of this, it is necessary to specifically study each natural space, as the spatiotemporal flow of hiker movement could vary considerably. This information could be very useful for predicting the pattern of movement of hikers for more efficient and safe management of this specific natural landscape [8,13,19,24].

To obtain this information, researchers have previously utilized devices such as the GPS [3,17,18,21,23,38,39], social networks such as Flickr and Wikiloc [13], photographs or videos [18,40], and a combination of these with questionnaires [8,15,17]. Nevertheless, the GPS device could record atypical values due to a positioning malfunction or mistake [17,18,22], as the GPS-based trails found in the devices themselves or downloaded from platforms such as Wikiloc may not coincide with the existing trails [13]. Furthermore, in some natural spaces, the connections via satellite for mobile phones or even the GPS signal from the different GPS devices available on the market today may not be precise enough. This lack of precision could be due to scarce coverage or lack thereof in the area, which may be interrupted by the existence of natural canyons or atmospheric conditions, for example [3,21,29,38,39]. Thus, natural areas could be found where it is impossible to perform an analysis of the hikers’ flow with this technology. However, technological innovation evolution allows for the use of eye-tracking devices, which are very valuable for extracting the type of information related with the study of movement patterns.

Eye-tracking devices have been broadly utilized in the area of sports [41,42], and are becoming more popular in other sectors related to marketing [43,44,45,46]. Eye tracking allows the direct and reliable measurement of eye movements in response to different situations or sensorimotor tasks to be performed [44]. Two types of devices can be differentiated: fixed and mobile [47]. With the latter, one can walk freely around any area while the gaze position is recorded as a video of the field of view. This allows the evaluation of the movement of the eye superimposed on the image of a real video through a positional cursor, while the subject who is wearing it performs any activity [42,48]. Visual attention studies within the tourism sector could be a key and novel technique [47], given that very valuable information about the different areas and elements of the trail that catch the attention of the hiker can be obtained with the use of this device.

Therefore, the main objectives of this study were to analyze (a) the spatiotemporal flow of the hikers in the El Caminito del Rey trail according to the hiker profile, and (b) the visual strategy followed by the hikers.

## 2. Materials and Methods

### 2.1. Study Area and Path

**Figure 1:**
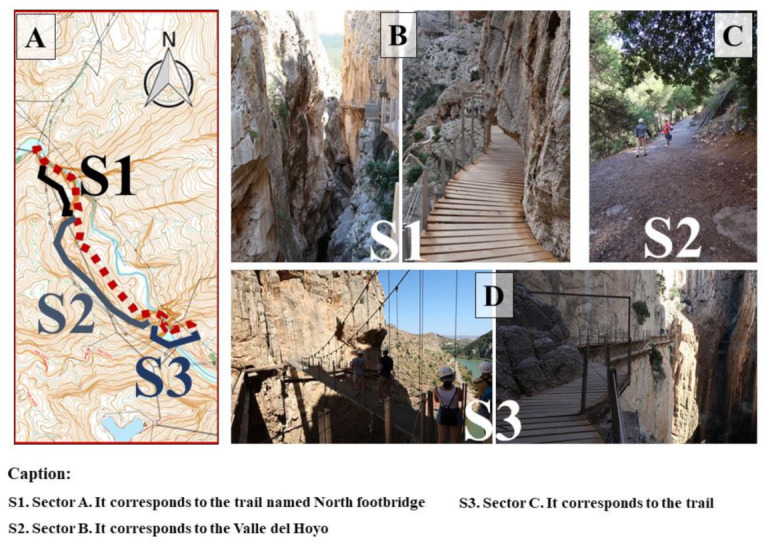
Location and sectors of the CR natural path. (**A**) Route of the El Caminito del Rey (CR) trail (**B**) Sector A; (**C**) Sector B; (**D**) Sector C.

The El Caminito del Rey (CR) trail is located in the south of Spain, in the province of Malaga within the municipality of Ardales (36°55′49.8″ N 4°47′04.4″ W). This nature setting has a length of more than 7 km, and is located within a complex natural environment, surrounded by reservoirs, mountains, passes, and valleys. Within the natural tourist attraction, its orography and design must be underlined. The trail is delimited by a mountainous area, surrounded by many gorges and passes on both sides of the trail. This makes it so that the trail travels along the walls, creating the need to build uncovered, hanging boardwalks on the side of these walls (Figure 1). This reconstruction of the trail by sections allows access to a natural space that is 3475 m in length, and which possesses not only great biodiversity, but also spectacular views, although the connection via satellite for mobile phones and GPS signals is nonexistent or bad due to the GPS signal bouncing off the walls along the boardwalks.

This trail is the object of our research, with its one-way linear route (not circular) that descends downwards from north to south. Due to its location and orographic characteristics, three semi-differentiated sectors can be observed throughout this trail (Figure 1A).

Sector A, named S1, corresponds to the trail named north footbridge, identified with its first boardwalk divided into two sections: The first is named Los Gaitanejos and the second is El Tajo de las Palomas. Its total length is 1172 m (Figure 1B). It is here where we find the access point and the start of the CR trail.

Sector B, named S2, corresponds to the Valle del Hoyo. Its total length is 1594 m (Figure 1C).

Sector C, named S3, corresponds to the second boardwalk, Los Gaitanes, also known as the south footbridge. Its total length is 710 m (Figure 1D). It is here where the CR path ends.

### 2.2. Design and Participants

A descriptive and cross-sectional design was utilized. The data collection took place between the months of February and June 2018. More specifically, the measurements corresponding to the determination of the visual strategy followed by the hikers took place in February (study 1). As for the measurements related with the spatiotemporal flow of the hikers, these were taken during the first week of June (study 2).

The sample was selected by using a non-random and consecutive sampling method, and it was composed of 1582 hikers. With this sample, a non-random convenience subsample was obtained for study 1. The subsample extracted was composed of 126 participants, of which 50.79% were men (NM = 64) and 49.20% were women (NW = 62). On the other hand, for the second study, the sample was composed of 1456 hikers, of which 48.3% were men (NM = 703) and 51.7% were women (NW = 753). In the case of the men, the mean age was 43.67 ± 16.19 years old, and for the women, it was 51.7 ± 14.78 years old.

Based on these previous research studies, the classification used to define the hikers’ profile was: group hikers guided by the CR staff (68%, NH1 = 990) (H1), group hikers guided by external operators (H2) (23.7%, NH2 = 345), and non-guided hikers (H3) (8.3%, NH3 = 121). All of the visitors were informed about the research study and provided their consent to participate. The study was conducted in accordance with the ethical principle of the Declaration of Helsinki for human research [49], and was approved by the institutional review boards of the participating universities.

### 2.3. Measurements and Procedures

#### 2.3.1. Study 1 Instruments

The main objective of the first study was to determine the visual strategy utilized by the hikers of the CR trail.

For recording this data, the Eye Tracking System (Tobii T60 Eye Tracker) monitoring system was utilized. The measurements were performed during a habitual visit to the trail. For this, the study researchers visited the CR installations during the complete trail hours set by the managing company from opening to closing time. In each of the days, various sessions and measurements were performed with the eye-tracking device. The hiker profile was not considered for the selection of the participating sample, so that every hiker was offered the possibility of participating to obtain general information on the visual strategy and more important points of interest throughout the trail, without the conditioning grouping factor.

Each of the participants was accompanied by a researcher who had specific training on the device utilized to guarantee the correct functioning of the monitoring system. Before hiking the trail, the participants completed a brief calibration exercise individually [33]. Following the recommendations provided in previous studies [42,44], the CR trail user was asked to hike the trail as planned in their itinerary. Lastly, the researchers did not previously select places to observe, and did not point the hikers to places they should be aware of during the hike.

##### Measurements

The visual behavior study was based on the places where hikers focused their attention to a greater extent. This is known as locations or fixations. For considering the action of fixation, a minimum duration of 200–300 milliseconds on the same location was established, based on previous research [47]. The greatest or least fixation duration on an image or determined area is related to a greater or lesser interest [44]. Furthermore, the fixation order performed by the subject, along with the probability of fixing the gaze to each location after observing each one of them, is a determining factor within gaze analysis [42]. More specifically, to obtain the information on the visual behavior of the CR hikers, the following measurements were collected [47]:

- Area of interest selected within the image (AI)
- Duration of the gaze on the AI in milliseconds (DG) and its percentage (%DG) related to the total exposure time of the image (fixations + saccades)
- Percentage of hikers who have observed the AI (PH)
- Percentage of hikers who visually revisited the AI (PHR); a re-visitor is defined as a visitor who comes back a second time or more to the AI
- Mean number of fixations of all of the participants who have observed the AI (NF)
- Thermal map/heat map coded with colors to determine the areas recorded with greater or lesser intensity during the eye tracking activity

#### 2.3.2. Study 2 Instruments

The main objective of this second study was to analyze the spatiotemporal flow of the hikers in the CR trail.

Firstly, the distances in meters of each area of the trail according to sectors were verified. To calculate the distance, an odometer was utilized (PCE instruments, model T593). The odometer is an instrument that allows the calculation of the distance travelled between two points when a GPS device loses its connection, which is a common occurrence within the trail studied. The distance is calculated thanks to a wheel that, when placed on the ground, rotates as the instrument is moved forward along the surface, so that the final distance is the product of the number of rotations and the perimeter of the wheel [50]. The odometer had the following characteristics (certified by the maker): tolerance < 0.02 and precision < 0.002.

##### Measurements

The results in meters obtained allowed us to identify the distances of the trail to be covered at the general and specific level. Thus, the total distance of the trail was 3472 m, divided into 1172 m for area S1, 1594 m for area S2, and 710 m for area S3.

Secondly, to quantify the number of users who hiked the CR trail, the mandatory-use helmets were numbered. These helmets were also identified with different colors according to the type of visit, so that, for the guided groups, the helmet was green, and for the non-guided hikers, the helmet was white.

**Figure 2:**
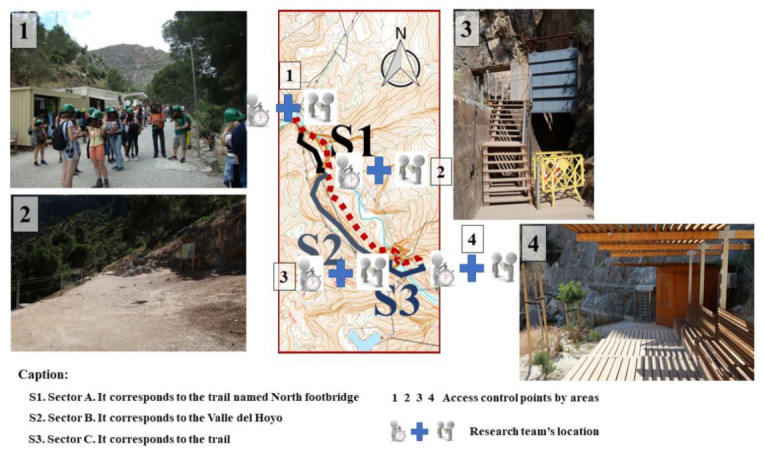
Research teams’ locations in the access points for the circulation of the hikers by areas.

Thirdly, work teams composed of two or three researchers were defined and placed at the access or exit points of each sector into which the trail was divided. In this way, four access control areas were identified (Figure 2).

Once the hikers arrived to the access point of the trail before the entrance, a researcher provided information and asked the hikers for participation, while another was in charge of providing numbered helmets to the participants and collecting basic demographic data, which could be used to pair the user with the specific helmet number. When the hikers accessed the trail, the third researcher recorded the hour of entry and the helmet number of each user. Each of the research teams at each of the access control points recorded the same information.

Lastly, the researchers visited the CR trail installations during the visiting open hours one day on the weekend (Saturday) on the first week of June. This decision was made due to aspects that are described below. Lera et al. [3] indicated that weather conditions affect the activities that take place outdoors. Nevertheless, the CR trail has certain particularities in its design, so that the trail is closed to the public when extreme heat, wind, or rain is expected. These same authors [3] also pointed out that the frequency of visits to nature areas is similar throughout the year. On the other hand, after consulting the sales records provided by the CR managing company, as expected, it was observed that there was a greater number of visitors during holidays and weekends. In light of this, a joint planning session was conducted with the CR managing company and the Malaga Council to ensure the maximum number of visitors for the study, so that, except for that day, an increase in the number of visitors by 21% was approved.

### 2.4. Data Analysis

The descriptive data are presented as means and standard deviations of the mean. For the eye tracking study, only a descriptive analysis was performed through the central tendency and dispersion indices of each of the variables considered. In the study of speeds recorded according to the trail sector, the Kolmogorov–Smirnov test was utilized to verify data normality. The data were not homogeneously distributed. Hence, non-parametric tests were applied during the statistical analysis. To detect differences in the speed scores according to the sector of the trail, the data were analyzed with the Friedman test. A Wilcoxon post hoc test was used to explore the differences among the conditions. For the data related to the travel speed scores according to sectors of the trail, as a function of the type of group visit, the Kruskal–Wallis test was used. A Mann–Whitney post hoc test was utilized to explore the differences among the three conditions. The effect size was calculated with Rosenthal’s r [51,52] and η2 [53] (0.1 to 0.3 (small), 0.3 to 0.5 (medium), and > 0.5 (large) effect). A significance level of *p* < 0.05 was accepted for statistical comparisons. The calculations were performed with SPSS Statistics for Windows, Version 24.0 (IBM Corp., Armonk, NY, USA). Lastly, the Monte Carlo simulation (number of simulations of different scenarios = 150) was utilized to simulate an environment through which to obtain experimental information about the variation in the density of circulation of the users according to the different sectors of the trail, with the source code available at https://github.com/fjbaron/CaminitoDelRey (accessed on 12 February 2021). The Monte Carlo simulation is a procedure based on a random simulation of the behavior of real variables used to analyze and predict their evolution [54].

## 3. Results

Due to the number of variables that were studied and analyzed, the results are shown step by step.

### 3.1. Study 1

**Figure 3:**
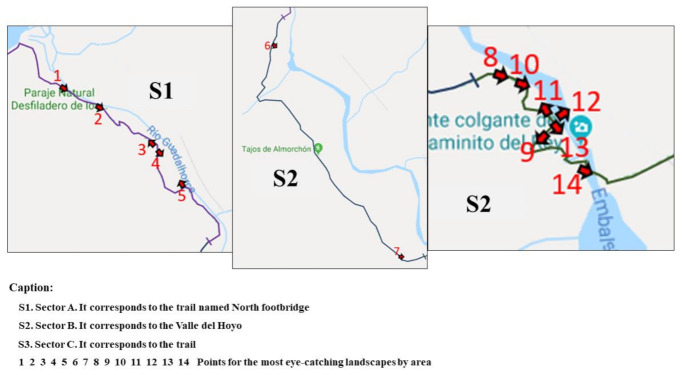
General location according to sector of all images analyzed through the eye-tracking device.

The general information about the images analyzed through the eye-tracking mobile device for each area of the trail is reported in Figure 3.

A clear predominance of landscapes that caught the hikers’ attention was observed for areas S1 and S3.

**Figure 4:**
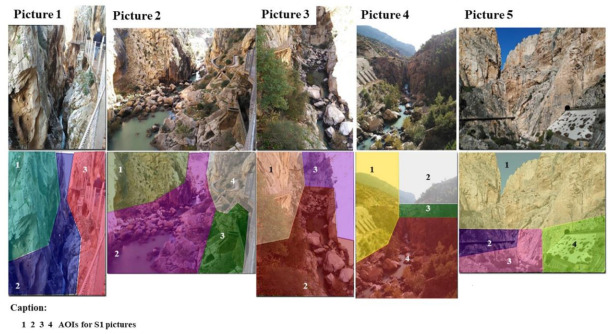
AI images for zone S1.

Next, concerning the S1 area, Table 1 shows the data collected for the landscapes that caught the hikers’ attention (Figure 4).

Even when the data revealed that the five images recorded in this sector obtained very high values for DG, these same results showed a greater DG in the landscapes from images 1 and 2. Regarding the values in the different images, only image 1 obtained a value of 100% of hikers who focused on all of the AIs. On the other hand, for image 2, AI4 obtained values for DG and NF that were higher than the rest of the AIs recorded in any other image from this sector (DG = 70.31sg; NF = 241.80).

**Figure 5:**
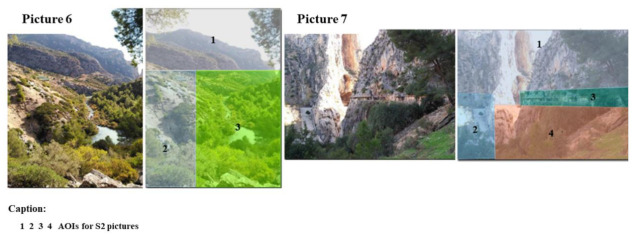
AI images for zone S2.

Next, regarding the S2 area, Table 2 shows the data collected for the landscapes that caught the hikers’ attention (Figure 5).

The results showed that these landscapes had a lower DG than the S1 area landscapes. More specifically, focusing on the S2 area, the AI3 for image 6 obtained the highest DG (78.34% and 80.44% higher than AI1 and AI2, respectively). For the last landscape that caught the hikers’ attention, image 7 obtained a higher DG compared to those recorded for image 6.

**Figure 6:**
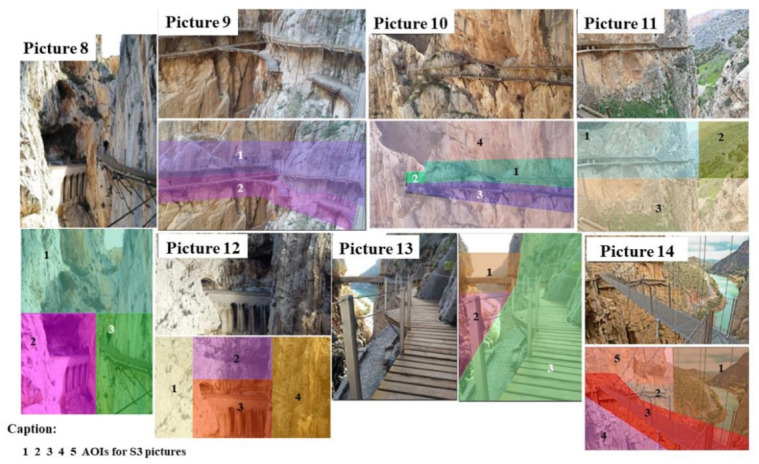
AI images for zone S3.

Table 3 presents the landscapes that caught the hikers’ attention for the S3 area (Figure 6).

As for the S3 area, the results showed higher DG values (10–13 s) than those recorded for the S2 area. Nevertheless, images 9, 13, and 14 should be noted, because they possessed AIs with DGs that were higher than the rest of the images. For image 9, 100% of the hikers focused their attention to the two AIs. However, in this landscape, AI2 obtained 26.29% more DGs than AI1. With regard to image 13, AI3 obtained a DG of 30.96 s. More specifically, this DG indicated an increase of 65.94% and 73.42% with respect to AIs 2 and 3, respectively. Furthermore, it was the only one whose AIs were viewed by 100% of the hikers.

**Figure 7:**
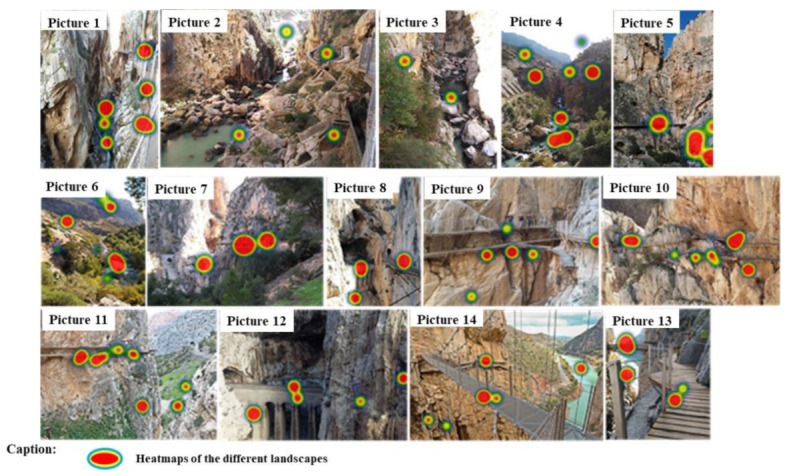
Heat maps of the different landscapes according to sector.

Lastly, the heat maps show a snapshot of the number and type of elements viewed, as well as the intensity with which each of the different landscapes was observed (Figure 7). The higher viewing intensities are shown as a red color, while the landscapes viewed with less intensity are shown with a green color. These heat maps corroborate the results presented above.

### 3.2. Study 2: General Description of the Hikers’ Frequency of Entry and Passage, and Speeds Utilized to Hike the El Caminito del Rey Path

The results revealed that the greatest hiker attendance was during the morning, with an accumulation of 76.6% of the hikers, whereas for the afternoon hours, the number of hikers decreased, with the percentage falling to 23.4%.

**Figure 8:**
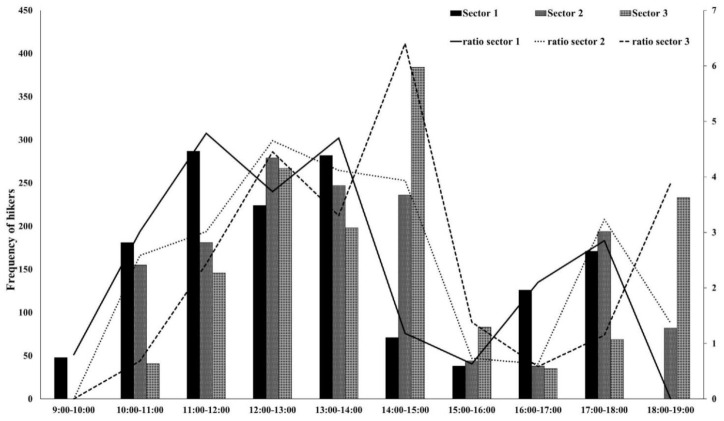
Frequency of hikers according to hours and sectors within the CR trail.

On the other hand, the hikers’ frequency and ratio according to time slot and sector showed variable behavior (Figure 8). More specifically, 72.12% of the total number of hikers who visited the trail hiked through zone S1 in the morning time slot (9 am–2 pm), while only 27.88% of them did so in the afternoon. The hours with the greatest hiker accumulation in this zone were from 11 am to 12 am (287 hikers (21.63%)), and from 1 pm to 2 pm (282 hikers (19.37%)). In area S2, it was observed that there was still a high percentage of hikers visiting this area in the morning time slot (59.20%). The time slots with the greatest hiker accumulation were those corresponding to the early afternoon hours from 12 am–3 pm (279 hikers (19.16%), 247 hikers (19.96%), and 236 hikers (16.21%), respectively). Lastly, regarding the S3 area, the trend was the opposite as that observed previously, because 55.22% of hikers visited the path in the afternoon. In this last sector, the greatest hiker attendance was observed in the time slot from 2 pm to 3 pm (384 users (26.37%)).

Next, the results found for walking speed according to sectors revealed significant differences (*p* = 0.05) (Table 4).

More specifically, significant differences were found for all of the comparisons between each of the walking speeds recorded (between S1 and S2 (Z = 26.06; r = 0.68, *p* = 0.000); between S1 and S3 (Z = −29.35; r = 0.77, *p* = 0.000), and between S2 and S3 (Z = −32.98; r = 0.86, *p* = 0.000)), with the walking speed from S2 being the highest one.

#### 3.2.1. Study 2: Walking Speeds Recorded when Hiking the El Caminito del Rey Trail as a Function of the Type of Visit

Concerning the average walking speeds according to sectors for each of the different visit types, significant differences were observed (*p* < 0.05) (Table 5).

Firstly, the hikers’ general walking speed revealed a significant difference between each visit type (*p* = 0.000, η^2^ = 0.11). More specifically, the H1 group exhibited a higher walking speed than the other groups. Secondly, according to the S1 area, significant differences were observed in the walking speeds among the hiker groups (*p* < 0.05, η^2^ = 0.02). Thus, the average speeds recorded for H1 and H2 were 11.83% and 14.19% higher, respectively, to that recorded for H3. In addition, H2 showed the highest speed overall. Along the same line, post hoc analyses indicated significant differences between each of the hiker groups for the trail in the S2 area (*p* = 0.000, η^2^ = 0.23). In this case, the H1 had increases in walking speeds of 17.59% and 34.05% compared to the speeds recorded for the H2 and H3 groups, respectively. Lastly, for the S3 area, post hoc analyses showed significant differences only for the average walking speeds recorded between the H1 and H2 groups (*p* = 0.000, η^2^ = 0.06) and H1 and H3 groups (*p* = 0.002, η^2^ = 0.13).

#### 3.2.2. Study 2: Simulation of the Distribution of the Users According to Trail Zones

**Figure 9:**
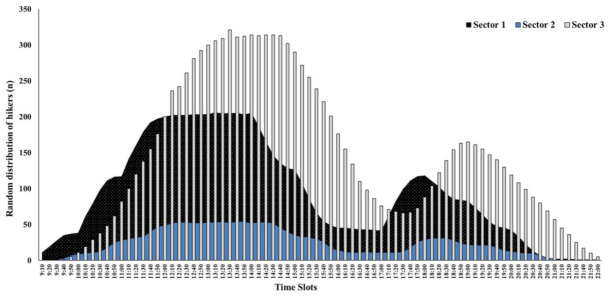
Monte Carlo simulation according to sector for the trail hikers.

Finally, the Monte Carlo simulation for a total of 150 different scenarios of hiker distribution according to zones can be observed in Figure 9. The first result observed in the simulation was the greater trend in the hiker accumulation for all of the sectors in the morning time slot. On the other hand, the simulation also allowed us to observe how the S2 area was the zone where there was the least accumulation of hikers (maximum peak of hikers accumulated recorded between 1 pm and 2 pm (N = 54 hikers)). Until 11:50 am, the S1 area obtained a probability of hiker accumulation (N = 197), which was higher than that recorded for the S2 area (N = 25) and the S3 area (N = 62). Another interesting finding was observed in the 12 pm time slot. A probability of 200 hikers was recorded for the S1 and S3 areas. In addition, concerning the S1 area, from 12:10 until 2:10 in the afternoon, a decrease in the probability of hiker accumulation was observed. This trend was prolonged to 2:30 for the S2 area (NS1 = range from 202 to 205 hikers and NS2 = range from 52 to 54 hikers). However, for the S3 area, a gradual increase was observed in the probability of hiker accumulation during these time slots, reaching a peak of 321 hikers at 1:30 pm. For this same area (S3), a probability of accumulation was observed for more than 300 hikers between the hours of 1 pm and 3:30 pm.

On the other hand, a decrease in the probability of hiker accumulation was observed in different sectors for the mid-afternoon hours, with this trend maintained for the S1 area from 2:10 pm to 5 pm (range: 186 to 41 hikers), the S2 area from 2:40 pm to 5:20 pm (range: 45 to 11 hikers), and the S3 area from 3:50 pm to 6 pm (range: from 201 to 67 hikers).

## 4. Discussion

The aims of the present study were to analyze: (a) the spatiotemporal hiker flow in the El Caminito del Rey trail according to the hiker profile, and (b) the visual strategy utilized by the hikers. This information can be used to better understand the spatial distribution of these hikers around the CR natural attraction. These movement patterns are very useful for the administrators of any park or natural attraction for more efficient administration and management of the human resources and equipment used during visitation hours of the trail [19,27,28,32], with the existing information about these types of recreational activities being limited [19,23,30].

Our results showed significant differences in the frequency of visits according to time slots, with the morning time slot (9 am to 2 pm) being the most dominant in hiker attendance. Barros et al. [13] found results that were partially similar, with a greater attendance of visitors to the natural areas in the midday time slots and closer to the late afternoon hours (11 am to 5 pm). This small variation in the time slots could be due to the opening and closing hours of each park or natural attraction, as well as the characteristics of the natural park or the trail themselves, or their location and driving distance to population centers or cities. In the end, all of these reasons will have an important and differential weight on the attendance and entry of the users, which indirectly affects the distributions and patterns of their visits [17,26]. These differences could also be due to the season of the year when the study was conducted. Lera et al. [3] found that the number of daylight hours had an influence on hiking activities in nature. In the summer, the daylight hours usually last 14 h, and in winter 10 h, resulting in the greater dispersion of users in the summer season. Meanwhile, in winter, the effect is the opposite, with a greater agglomeration of users in these middle time slots and the beginning of the evening due to the earlier sundown time. This is a determining factor that could explain the behavior and flow differences between the natural attractions.

Additionally, in our study, it was observed how the time slots with the greatest hiker agglomeration varied as a function of the sector of the trail studied. Orellana et al. [21] defined the concept of visitor flow as the aggregated movement of people who visit different places in a generalized sequence, independently of the route followed by each individual. In this sense, through the Monte Carlo simulation, a simulation and prediction of the position of the users according to sectors was provided, which allowed us to obtain a generalized view of the possible user location and flow. As opposed to findings from other studies, which indicated great diversity in the flow of visitors or the detour of the hikers away from the official route [17,21,26,39], our research results indicated a more fixed and pre-determined pattern of movement as a result of the existence of a single point of entry and exit in a single trail direction. In light of the above, we can state that the distribution will depend on the specific physical characteristics of the trail hiked [3,17] and, of course, the timetables set by the guides [10]. Furthermore, these findings do allow for identifying the hours of maximum concentration according to zones throughout the day, which is very useful for management companies and administrators of the area. Based on this, it is possible to discover if the flow of entry and agglomeration (hiker accumulation) according to sectors is adequate, or a greater redistribution is needed to better monitor and experience the visit, which would guarantee the safety of the hiker, as well as better environmental preservation of the area visited [1,8,11,13,14,21,24,32].

On the other hand, D’Antonio and Monz [18] found that, as opposed to what was expected, the hikers tended to agglomerate more when the area was more frequented, and there was a greater dispersion when the areas were not as frequented [14,19]. The explanation of this agglomeration trend in very specific areas is simple. The areas where agglomeration occurs are probably areas where there is something interesting or different to observe within the natural landscape of the trail. As stated in the ART, observing an attractive natural landscape with great biodiversity will provoke a great fascination in a person [35,36,37]. The results found in our study are in agreement with this statement, as the zones in sectors 1 and 2 were the ones where a greater number of images was recorded, which generated interest in the hiker. If these photographs are examined, we can corroborate how the landscapes that were more interesting were specifically identified with water, the river running through the gorges, the hanging bridge, or the boardwalk anchored to the walls through which the trail goes. Each of these compositions of the landscape has very specific and different characteristics, which differ from the routine composition that can be observe in an urban setting or a green area close to these population centers [12,13,14,15,17,33,36,37]. As a consequence, all of the hikers tended to pause in these areas for a specific amount of time, resulting in their agglomeration [19].

On the contrary, those areas with a low ratio of accumulated hikers can be the result of the lack of landscape attractiveness and the non-occurrence of that “call-effect” for hikers [15,17], as shown by the results from our study, where, in the zone named sector 2, only two interesting landscapes were recorded throughout its length. In fact, when analyzing the photographs of the landscapes that caught the attention of the hikers, the composition once again shows water, the gorges, and the structure of the boardwalk. These landscape areas do not truly belong to the part of the trail that goes through the valley, where the landscape is composed of a green area with trees on its sides. This seems not to be in agreement with findings from other studies, which point to the presence of trees as a determinant factor for capturing the attention of the hiker [12,25]. Nevertheless, as affirmed by Chhetri et al. [15] and Ried et al. [36], the perception of the landscape is a complex cognitive construction, in which the hiker will constantly change his or her evaluation of the scenic beauty throughout the hike as a result of the different attributes that comprise the scene. Based on this, if the landscape as a whole includes relevant physical characteristics and aesthetic beauty (such as lakes, waterfalls, or constructed elements that are in harmony with the nature viewed, which facilitate access to areas and/or landscapes that are difficult to access and beautiful), it is not strange that these forested areas are less interesting to the hikers [14,15,36].

On the other hand, just as the present study, Meijles et al. [29] found differences in the average walking speeds as a function of the hiker’s profile and motivation. However, the speeds recorded by these researchers were much higher in all of the groups compared to those obtained in our study, except for the H1 hiker group in the S2 area, where the average speeds were very similar. The possible explanation for these differences may reside in the orographic profile of the trails analyzed in both studies. As mentioned by Meijles et al. [29], the speed depends on the trail being more or less flat, or having good access throughout its entirety. In the specific case of the CR trail, zones S1 and S3 are characterized by the narrowing of the boardwalk and the existence of parts of the trail with stairs that must be climbed or descended. These characteristics of the layout of the trail, although not limiting entry to anyone according to their level of physical conditioning or ability, could be the reason behind the slowing down of the walking speed, especially when groups are encountered in these areas. Furthermore, it should be remembered that a great part of the attraction of these types of activities in nature is based on the potential for the exploration of remote locations [14,17,30,31,40]. In general, the S1 and S3 zones possessed a greater number of scenic landscapes that caught the hikers’ attention, thus resulting in lower average walking speeds. Orellana et al. [21] associated a reduction in speed to hikers stopping in areas where they found something interesting to observe. This could explain the results found in our study. Therefore, the geographical context where the hike takes place is also a determining factor when looking for an explanation of the existing movement patterns [14,17,18,21,30,31,40]. This is even more important in an area such as the CR trail, where the existing orographic differences of the terrain between each sector of the hike are evident.

Another possible explanation for the differences found in the average walking speeds could reside in the nature of the visitor groups themselves. In our research study, only a small percentage of hikers opted for being in non-guided groups. That is the reason why the walking speed was dependent on two main variables: the guide and the heterogeneity in the number and interests of the hikers who came together as part of the same hikers’ group. More specifically, the greater the size or the number of children or older individuals in the group, the slower the walking speed [27,29,30,31], which could justify the results found here. In addition, Boller et al. [14] attested that the selection of different visitor packages when hiking the trail plays an important role, as the hiker could hire guided visits through private or tourist companies, audio guides, or, on the contrary, could hike the trail alone. There is no doubt that, for a non-guided hiker, it is he or she who sets the pace and defines the places where he or she wants to spend more or less time. However, in a guided hike, it is the guide who sets the pace and walking speeds [27,30,31], which could vary due to diverse reasons. These reasons could be the in-depth knowledge of the place or the structure of the visit, which could be based on a more complex tourism agenda that relies on a strict schedule to follow based on the pack of activities chosen, and in which trail hiking is just another activity. This could explain the results found here, in which the guided groups had higher average speeds than those that were non-guided. The guided tours conducted by the CR staff had the highest average speeds in all of the sectors. Explanations for this could vary, as it is possible that there is a pre-established chart that determines the guiding times and resting time of the personnel, or this could perhaps be due to the staff’s greater knowledge of the area. Independently of the reason, knowing this information will allow for a more efficient and direct structuration and organization of the spatiotemporal flow of the hikers.

## 5. Conclusions

In general, the results found in this study showed that there was a greater hiker attendance at the CR natural attraction in the early morning hours. Furthermore, a clear difference was detected in the trend of hiker accumulation according to the zones into which the trail was divided. This information is highly relevant, as it could be used by the trail managers for the equilibrated design of the visits according to zones and time slots. On the one hand, with the data obtained through the Monte Carlo simulation, it was possible to verify how the hiker distribution and density throughout the trail varied as a function of the different zones. More specifically, it was verified that zone S2 maintained its probability of having the lowest hiker accumulation at all time slots, so that it could be a key area for the management and regulation of the flow of visitors, and for facilitating the resting of the guides within the CR. Furthermore, for zone 2 of the trail, the park managers could create additional measures to increase the interest of the visitors, for example through the placement of information panels about the flora and fauna of the area, or recreation or rest areas with benches that will allow the hikers to observe the landscape in this part of the trail. These measures could provoke changes in the spatiotemporal behavior of the visitors, which could result in the redistribution of the hikers throughout the trail. Through the use of eye-tracking devices, it was verified that sectors 1 and 3 had landscapes that were more attractive, which caught the attention of the hikers to a greater degree, as well as increased the trend in the number of visitors recorded through the Monte Carlo simulation. However, at this point, we should not forget that the S1 and S3 parts of the trail have elements such as stairs or narrowing of the trail that are common, and could be important elements that can limit and provoke the changes observed in the time needed to hike the trail, as well as the hiker conglomeration trends in specific parts. Therefore, the combination of both elements could be responsible for the different speeds recorded throughout the hike in each sector and depending on the type of hiker. This information should be considered when establishing the starting time of guided groups and the time they can spend in the trail according to sector.

To our knowledge, this is the first study that considers the hiker circulation patterns combined with eye tracking. The information recorded in this study is of vital importance when effective management strategies for the hikers’ safety need to be created. Furthermore, this information could be relevant for the evacuation plans in case of an emergency, as well as for designing more efficient communication strategies through the localization of zones that are more optimal and adequate for the placement of information about the natural environment that is visited.

In addition, these results will allow us to more specifically discover the hikers’ movement pattern, as it allows for measuring different aspects that condition their behavior throughout the trail, and it could allow better distribution and time management of the hiker groups that is tailored to the real visiting times. On the other hand, the design of strategies adapted to the reality of the CR trail itself could lead to improvement in the hikers’ experiences, as it will allow the administrators to evaluate the natural trail’s popularity according to its different zones to thereby improve the distribution of the personnel as a function of this, which is a direct reflection of a more efficient and safe management of the hiker in the long run.

Finally, this study has some limitations that should be noted. Aspects such as the weather were not considered (such as the air temperature or season), and information was not registered related to the expectations and satisfaction of the hikers, or the internal mechanisms of the guided groups. Furthermore, no studies were found that utilized a similar methodology, so it would be interesting to replicate this research study in the future, having in mind the previously-mentioned elements. Lastly, in the present study, we did not take into account the segmentation of the results obtained in the eye-tracking study as a function of the hiker profile (H1, H2, or H3), and this line of research should be considered in future studies.

## Figures and Tables

**Table 1:** Fixations and gaze by AI for S1.

		AIs_SQ	DG (sg)	%DG	PH (%)	PHR (%)	NF
S1	IMAGE 1	1	12.55	12.16	100	80	45.00
		2	30.39	32.54	100	80	102.25
		3	24.43	28.31	100	80	95.00
	IMAGE 2	1	10.54	6.11	100	100	34.60
		2	39.83	25.24	100	100	135.20
		3	3.85	1.89	80	60	13.80
		4	70.31	39.49	100	100	241.80
	IMAGE 3	1	10.52	25.46	100	60	27.75
		2	4.59	7.83	75	60	15.75
		3	8.62	17.51	100	60	27.75
	IMAGE 4	1	6.51	5.81	100	100	22.40
		2	1.70	2.98	80	40	6.40
		3	3.07	6.16	80	60	10.40
		4	13.84	22.20	100	100	56.40
	IMAGE 5	1	7.99	15.8	66.67	20	26.67
		2	4.54	22.03	100	40	12.67
		3	4.93	26.65	100	60	18.67
		4	4.77	14.82	100	40	17.67

Legend: S1 = sector 1; AIs = areas of interest; SQ = sequence of arrivals to the AI; DG = duration of the gaze with respect to the total exposure time of the image; % = percentage; PH = percentage of hikers who observed the AI; PHR = percentage of hikers who revisited the AI; NF = fixation number.

**Table 2:** Fixations and gaze by AOI for S2.

		AIs_SQ	DG (sg)	%DG	PH (%)	PHR (%)	NF
S2	IMAGEN	1	2.12	11.48	66.67	60	8.17
		2	1.92	18.84	83.33	40	7.50
		3	9.80	31.34	66.67	60	32.50
	IMAGEN	1	4.90	13.72	75	60	15.50
		2	3.57	8.77	100	80	12.75
		3	12.26	25.66	100	80	44.00
		4	10.72	20.86	100	80	37.75

Legend: S2 = sector 2; AIs = areas of interest; SQ = sequence of arrivals to the AI; DG = duration of the gaze with respect to the total exposure time of the image; % = percentage; PH = percentage of hikers who observed the AI; PHR = percentage of hikers who revisited the AI; NF = fixation number.

**Table 3:** Fixations and gaze by AOI for S3.

		AOIs_SQ	DG (sg)	%DG	PH (%)	PHR (%)	NF
S3	IMAGE 8	1	1.91	4.75	50	40	6.50
		2	11.56	55.42	100	60	40.00
		3	9.74	28.69	75	40	32.50
	IMAGE 9	1	12.44	34.04	100	80	42.50
		2	16.88	45.18	100	80	57.00
	IMAGE 10	1	11.77	28.3	100	80	41.75
		2	10.28	21.5	100	60	31.75
		3	10.30	17.79	100	60	32.75
		4	0.32	0.86	50	0	0.75
	IMAGE 11	1	4.04	37.47	100	80	15.50
		2	1.72	11.66	50	40	5.00
		3	3.14	24.67	100	40	12.00
	IMAGE 12	1	2.29	7.94	75	60	6.50
		2	0.92	2.31	50	40	3.75
		3	10.69	32.32	100	80	35.50
		4	6.45	22.99	100	80	24.75
	IMAGE 13	1	10.54	17.03	100	80	36.25
		2	8.23	10.68	100	80	29.00
		3	30.96	38.18	100	80	107.00
	IMAGE 14	1	12.81	10.89	75	0.6	43.25
		2	10.67	7.81	75	0.4	36.50
		3	24.57	26.36	100	0.4	19.75
		4	5.79	3.92	75	0.4	19.75
		5	0.00	0.00	0	0	0.00

Legend: S3 = sector 3; AIs = areas of interest; SQ = sequence of arrivals to the AI; DG = duration of the gaze with respect to the total exposure time of the image; % = percentage; PH = percentage of hikers who observed the AI; PHR = percentage of hikers who revisited the AI; NF = fixation number.

**Table 4:** Descriptive data for the time and speed utilized for hiking the trail.

	Speeds (m/min)				
	M	SD	χ^2^	df	*p*
S1	31.86 ^b,c^	±9.38	3087.219	3	0.000 **
S2	42.31 ^a,c^	±11.43			
S3	22.40 ^a,b^	±4.70			
General	31.66	±6.60			

Legend: m = meters, min = minutes, S1 = sector 1, S2 = sector 2, S3 = sector 3, significant post hoc differences between groups: ^a^ S1, ^b^ S2, ^c^ S3, ** *p* < 0.0.

**Table 5:** Mean travel speed utilized to hike each of the sectors that divide the CR trail.

Mean Speed According to Sections (m/min)							
	Type of Visit	M	SD	χ^2^	df	ρ	*p*
S1	H1	31.96 ^b,c^	±10.49	28.756	2	−0.018	0.000 **
	H2	32.84 ^a,c^	±6.20				
	H3	28.18 ^b,c^	±5.94				
S2	H1	45.50 ^b,c^	±11.65	337.069	2	0.462	0.000 **
	H2	37.49 ^a,c^	±7.32				
	H3	30.01 ^a,b^	±2.93				
S3	H1	22.94 ^b,c^	±4.96	30.463	2	0.14	0.000 **
	H2	21.53	±3.99				
	H3	20.55 ^a^	±3.38				
General	H1	32.73 ^b,c^	±7.45	168.174	2	−0.286	0.000 **
	H2	30.46 ^a,c^	±2.89				
	H3	26.29 ^a,b^	±2.00				

Legend: m = meters, min = minutes, S1 = sector 1, S2 = sector 2, S3 = sector 3, significant post hoc differences between groups: ^a^ Type of visit H1 = group hikers guided by the CR trail staff, ^b^ type of visit H2 = group hikers guided by external operator, ^c^ type of visit H3 = non-guided hikers, ** *p* < 0.01.

## Data Availability

The authors confirm that the data supporting the findings of this study are available within the article and/or its Supplementary Materials.

## Supplementary Materials

The following are available online at https://www.mdpi.com/1660-4601/18/4/1809/s1, Data sheet.
